# The Optical Property of Core-Shell Nanosensors and Detection of Atrazine Based on Localized Surface Plasmon Resonance (LSPR) Sensing

**DOI:** 10.3390/s140713273

**Published:** 2014-07-23

**Authors:** Shaobo Yang, Tengfei Wu, Xinhua Zhao, Xingfei Li, Wenbin Tan

**Affiliations:** 1 State Key Laboratory of Precision Measuring Technology and Instruments, Tianjin University, Tianjin 300072, China; E-Mails: yangskyle@tju.edu.cn (S.Y.); wtf@tju.edu.cn (T.W.); 2 School of Mechanical Engineering, Tianjin University of Technology, Tianjin 300131, China; E-Mail: xinhuazhao@tjut.edu.cn; 3 School of Mechanical Engineering, Tianjin University of Commerce, Tianjin 300134, China; E-Mail: twb@tju.edu.cn

**Keywords:** core-shell, nanosensors, atrazine, LSPR

## Abstract

Three different nanosensors with core-shell structures were fabricated by molecular self-assembly and evaporation techniques. Such closely packed nanoparticles exhibit fine optical properties which are useful for biochemical sensing. The refractive index sensitivity (RIS) of nanosensors was detected by varying the refractive index of the surrounding medium and the decay length of nanosensors was investigated using a layer-by-layer polyelectrolyte multilayer assembly. The results showed that the thickness of the Au shell plays an important role in determining the RIS and the decay length. A system based on localized surface plasmon resonances (LSPR) sensing was constructed in our study. The core-shell nanosensors can detect 10 ng/mL atrazine solutions and are suitable for pesticide residue detection.

## 1. Introduction

Noble nanoparticles such as gold and silver have attracted much attention both in the biosensor and disease diagnosis fields during recent years due to their unique optical properties [1–3]. One of the most interesting properties is known as localized surface plasmon resonances (LSPR), which results from the collective oscillation of the free electrons excited by an incident electromagnetic wave [4,5]. It is now well established that the properties of nanoparticles are strongly dependent on the nanostructure shape, size and composition [6]. The plasmon resonances associated with noble metal nanostructures create sharp spectral absorption and scattering peaks, as well as strong electromagnetic near-field enhancement near the nanoparticles' surface, and the position of that is very sensitive to tiny variations in the local environment of the nanoparticles [7]. Therefore, sensors based on LSPR sensing are suitable for molecular detection at low concentration, especially in chemical and biological sensing, which is becoming a research hotspot.

The commercialized SPR sensors have many advantages as optical biosensors in sensitivity and real time detection of biomolecular interactions, which allow them to be widely applied in practice. However, LSPR sensors that take advantage of the SPR sensing principle and the characteristics of binding interactions have emerged as a novel sensing tool which has been used to monitor a variety of processes, including antigen-antibody interactions, and DNA hybridization [8,9]. They have a great advantage in the simplicity of the detection system, that is, input as light and output as transmitted/reflected light intensity, allowing for small sensing devices [10,11]. The most important advantage is that compared with SPR sensors or other sensors, to a certain degree LSPR sensing can offer high sensitivity for low-abundance analysis without labels because of the highly localized electromagnetic field near the nanoparticle surfaces that is directly associated with the limit of detection and the sensitivity of nanosensors.

The LSPR sensing principle mainly depends on the spectral shifts caused by the surrounding dielectric environmental changes when adsorbents bind to the surface of the nanoparticle. The optical responses of LSPR sensing are determined not only by the refractive index of the medium, but also by the distance from the metal surface. The electromagnetic field surrounding the nanoparticles is not uniformly distributed, but is a function of the distance from the metal surface, and the evanescent plasmon field away from the nanoparticles' surface decays exponentially [12]. The decay, which depends on the type of metal and physical parameters of the nanoparticles, can be best described via the following equation [13]:

(1) R=mΔn[1−exp(−d/l)]

where R is the wavelength shift or intensity change; m is the refractive index sensitivity, that is, resonant peak shift or intensity change per refractive index unit (RIU) change; Δn is the change in refractive index (RI) of the surrounding medium, which is usually caused by adsorbents; d is the thickness of the binding layer, and l is the effective plasmon decay length. From the above equation it is found that the plasmon response of metal nanoparticles decreases exponentially far away from the nanoparticle surface. Researchers have found that the enhanced electromagnetic fields in LSPR are strongly localized, with decay lengths of several nanometers in any direction normal to the nanoparticle surface. Further, the decay length of LSPR can be tuned by varying the nanoparticle size, shape, and composition [14–16].

We have tried to fabricate ordered arrays of non-close-packed spherical polystyrene particles which can be prepared to exhibit precisely controlled diameters and interparticle distances. The size of PS spheres can be efficiently reduced with plasma etching, and the surface topography can be manipulated by controlling the initial PS sphere size and the time of plasma exposure. These non-close-packed LSPR sensors have great advantages of tunable size and interparticle distances of the PS spheres, which can lead to different LSPR resonant peaks and can be suitable for special sensing applications such as surface wettability or hydrophobicity. Some experiments show that the uniformity in size, interparticle distance and surface topography is exceedingly inhomogeneous. The plasma etching parameters were difficult to determine for us to satisfy our experimental requirements. Reproducibility is extremely important for a sensitive biosensor, so we chose a core-shell structure for sensing. Co-Ag nanoparticle core-shell structures have been synthetised [17]. Researchers found a strong enhancement of the magneto-optical Faraday rotation in all-metal core-shell Co-Ag nanoparticles attributed to localized surface plasmon resonance, which may enable the design of nanostructures for modulated sensing, imaging of magnetic fields and miniaturized magneto-optical devices, but compared to Au-shell structures, Ag-shell ones are easily oxidised and unstable. In addition, Au can react with sulfhydryl compounds forming stable and solid Au-S bonds, which offers a way to combine with more molecules for surface modifications suitable for sensing.

In our quest for effective and stable sensors that would perform well in experiments, in this study we fabricated three different sensors by varying techniques and parameters. Next, we further investigated the optical properties of the nanosensors, as well as their sensing performance, such as the RIS and the decay length, and the thickness of Au shell and Au film substrate is considered to be a reasonable method to tune the properties of the RIS and the decay length which play an important role in analytical and biological applications. Finally, we provide an overall and reasonable idea to evaluate the sensing capability in a LSPR sensing procedure.

## 2. Experiment Section

### 2.1. Materials

The substrates used were JGS1 quartz glass slides (10 mm × 10 mm, supplied by Jinghe Optical Instrument Factory, Jiangsu, China; polystyrene spheres (PS: mean diameter: 590 nm, Bangs Laboratories Inc., Fishers, IN, USA); Gold (99.99%, Shuosong electronic technology limited company, Shanghai, China); polyallylamine hydrochloride (PAH, 58 kDa, Sigma-Aldrich, St. Louis, MO, USA); polystyrene sulfonate, sodium salt (PSS, 70 kDa, Sigma-Aldrich); sodium chloride (99.5%, J & K, Beijing, China); H_2_SO_4_ (98%), H_2_O_2_ (30%), alcohol (99%) and ammonia solution (25%) were received from Heowns (Tianjin, China); 4,4-dithiodibutyric acid (DDA) 1-ethyl-3-[3-dimethylaminopropyl] carbodiimide hydrochloride (EDC), monoclonal mouse anti-IgG atrazine antibody (ABIN234335, purified liquid) was purchased from Antibodies-Online GmbH (Aachen, Germany) and N-hydroxysuccinimide (NHS) was acquired from Sigma-Aldrich (Schnelldorf, Germany). Atrazine, phosphate-buffered saline and high-purity nitrogen obtained locally. All solutions were prepared using triply distilled water.

### 2.2. Apparatus

UV-VIS-NIR light sources (DH-2000-BAL, Balanced Deuterium Tungsten Source, 210–1700 nm, Ocean Optics. Inc., Dunedin, FL, USA), optical probes (Premium 600 um Refl. Probe, VIS/NIR), Spectrometers (Maya2000 Pro, Ocean Optics. Inc.), thin films (WS-1, diffuse reflectance standard, PTFE, Ocean Optics. Inc.), pulp refiner (KW-4A model, Chinese Academy of Sciences, Beijing, China), SEM (SSX-550, Shimadzu, Kyoto, Japan), ion sputtering instrument (SBC-12, Kyky Technology Co. LTD., Beijing, China), Peristatic pump (Masterflex L/S, Cole-Parmer, Vernon Hills, IL, USA), Flow cell and trestle table.

### 2.3. Detection System

**Figure 1.**
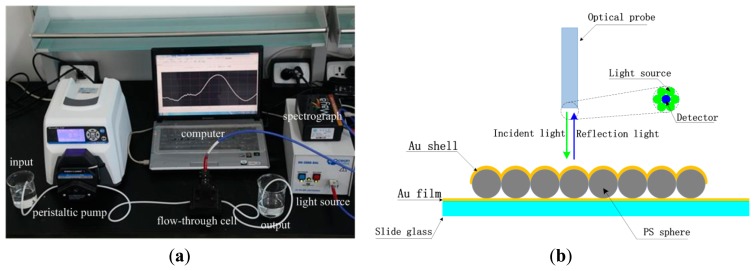
(**a**) A photograph of the detection system; (**b**) A schematic diagram of core-shell nanosensors in reflective mode.

The solution provided by peristaltic pump flowed into the flow cell from the left beaker, and the water solution was collected in the right beaker shown in Figure 1a. The input light produced by the light source shined vertically the surface of the nanosensors by optical probe and the reflected signal was obtained by the spectrograph, and a computer processed the reflected signal to produce an output reflectivity or extinction spectrum. Figure 1b is a schematic diagram of core-shell nanosensors in reflective mode. The core-shell nanosensors were fixed in the flow-through cell. The reflectivity is equal to the reflective light intensity divided by incident light intensity. It is dimensionless number and is the applied normalization method in this paper.

### 2.4. Preparation of Core-Shell Nanosensors

The LSPR-based nanosensor is a core-shell structured nanoparticle layer substrate constructed of monolayers of closely-packed nanoparticles [18,19]. PS nanospheres were used as the “core” of the nanochip, and a gold layer was applied with the “shell”, which was thermally deposited onto the core surface shown. To form closely-packed monolayerws, all glass slides were cleaned for 1 h in freshly prepared “piranha” solution (H_2_O_2_:H_2_SO_4_, 3:7 by volume. *Caution*: “piranha” solution is extremely corrosive and boils upon mixing). This was followed by a rinse in ultrapure water and sonication in a mixed NH_3_:H_2_O_2_:H_2_O = 1:1:5 (by volume) solution for 1 h to obtain a hydrophilic surface. Finally, all glass slides were washed in ultrapure water in which they were stored until use. Next, a drop of mixture solution from a commercially purchased suspension of polystyrene spheres with a diameter of 590 nm at a concentration of about 10% and ethanol by volume 1:1 was spin-coated on the slide at a typical rotation rate of 2500 rpm for 10 s. The slide was slowly immersed into the container with ultrapure water. After the monolayer of PS in water was stable, the monolayer of PS was absorbed on the other slide. Au shell and film were formed by using an ion sputtering instrument (conditions: vacuum: 10 Pa; current: 10 mA; rate: 1 nm/s).

### 2.5. Preparation of the Polyelectrolyte Multilayers by Layer-by-Layer Assembly

Prior to the layer-by-layer (LbL) assembly, the nanosensors were washed 20 min in ethanol and dried under a nitrogen stream. We used layer-by-layer deposition to sequentially deposit polyelectrolyte layers of opposite charge on immobilized gold film [20]. The LbL procedures were carried out as follows: first, the nanosensor was immersed 15 min in a solution of the positive polyelectrolyte poly (allylamine hydrochloride) (PAH) with a concentration of 1.0 mM, then washed in ultrapure water. Next, the nanosensor was washed successively in water and 0.1 M sodium chloride solutions to get rid of superfluous PAH, then it was immersed 15 minutes in a negatively charged polyelectrolyte solution of poly(styrenesulfonate) sodium salt (PSS) with a concentration of 1.0 mM. A number of polyelectrolyte bilayers were sequentially deposited on the slide in the similar manner. After the deposition of each polyelectrolyte layer, the slide was washed three times and dried under a nitrogen stream, and the extinction spectrum was measured by the detection system.

### 2.6. Biofunctionlization of Core-Shell Nanosensors

The core-shell nanosensors were washed with ethanol/acetone mixture (volume ratio of 1:1) in an ultrasonic bath at room temperature for 30 minutes and dried under a nitrogen stream, followed by immersion in 1 mM DDA solution for 12 h at room temperature. Then, the core-shell nanosensors were washed with ethanol to remove the excess thiol and dried with nitrogen. Further, the terminal carboxyl groups of the thiolated surface were activated with mixed EDC/NHS (0.4 mM/0.1 mM) aqueous solution at room temperature for 50 min. Next, anti-IgG atrazine monoclonal antibodies diluted in PBS buffer (100 μL of 100 μg/mL) were incubated with the activated gold shell at 4 °C for 12 h. After PBS rinsing, a BSA solution (0.1 mg/mL) was used to block the non-specific binding sites of the antibody-modified NPs. Finally, various atrazine dilutions with concentrations ranging from 10 ng/mL to 1 μg/mL diluted in PBS were incubated with antibody-modified nanosensors at 4 °C for 3 h to complete the antibody-antigen immunoreactions. The LSPR responses were recorded after each (bio) functionalization step by the detection systems.

## 3. Results and Discussion

### 3.1. The Core-Shell Nanosensors

**Figure 2.**
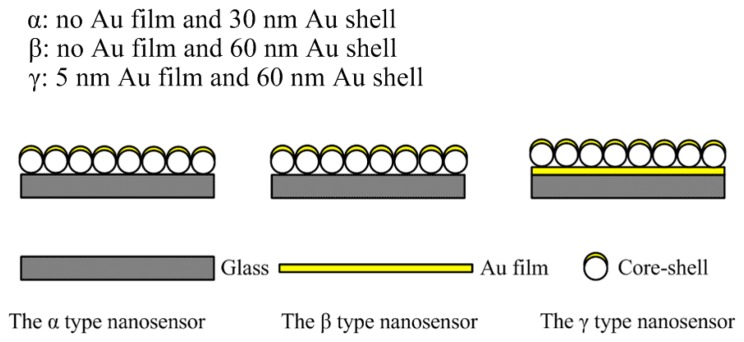
The structure of the three types of nanosensors.

In our study three different LSPR core-shell nanosensors (shown in Figure 2 and called for convenience *α, β, γ* in following sections) were fabricated by using spin-coating and Au deposition technology. There are two key points in the process of producing the sensors: (1) the concentration of the PS must be appropriate in the spin-coating step, otherwise the PS will clump or distribute loosely and not be able to form a uniform large-area monolayer; (2) the slide dipping into the water must be extremely slow, because the connection strength between PS particles is very weak. Figure 2 shows the three different LSPR nanosensors. The *α* type sensor was a 30 nm Au shell on a 590 nm monolayer of closely-packed PS supported on a glass slide. The *β* type sensor was a 60 nm Au shell on the 590 nm closely-packed PS monolayer supported on the glass slide. The *γ* type sensor was a 30 nm Au shell on a 590 nm monolayer of closely-packed PS supported on a 5 nm Au film over the glass slide.

**Figure 3.**
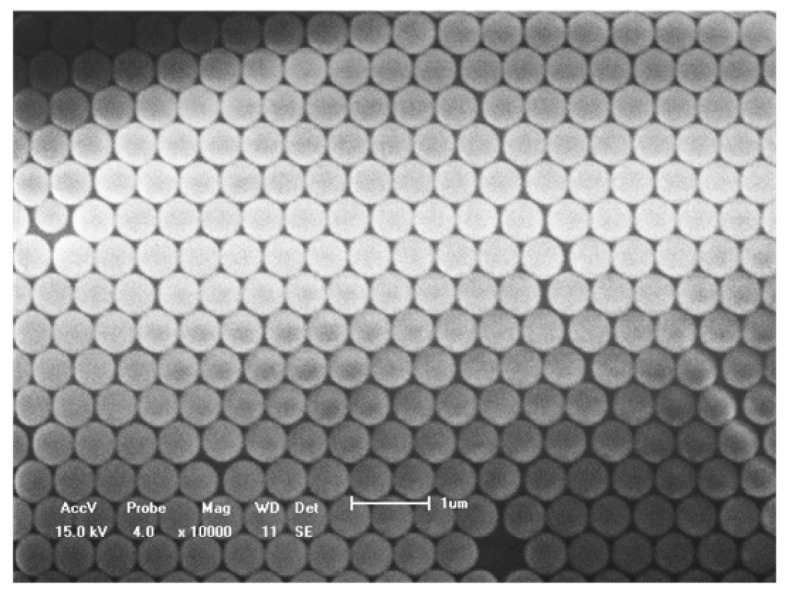
SEM image of closely-packed 590 nm PS supported on a glass slide.

Figure 3 is the SEM image of the PS monolayer on the glass slide measured by scanning electron microscope (acceleration voltage: 10 kV, magnification: 18,000× and working distance: 10 mm). It is apparent that the nanoparticles are closely-packed with each other, which provides an effective support for the stability and repeatability of the nanosensors.

**Figure 4.**
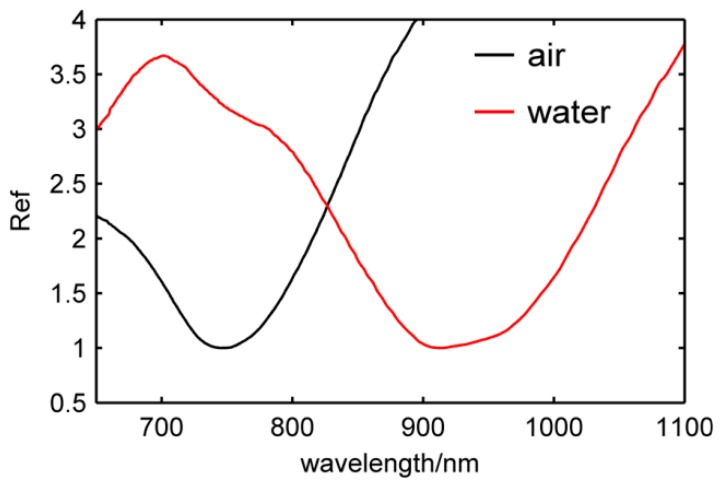
The reflectivity value (Ref) of *α* type nanosensor in air and water.

The LSPR peak shifts from 750 nm to 905 nm as shown in Figure 4 when the surrounding medium changes from air to water. This shows that the nanosensor is exceedingly sensitive to the RI of the surrounding medium, so this sensor can be used for high sensitivity sensing.

### 3.2. Bulk Refractive Index Sensitivity and Figure of Merit of the Nanosensors

The simplest sensing application of nanosensors is to detect changes in the bulk refractive index of their environment through shifts in the LSPR peak wavelength. Because LSPR sensing is based on spectral peak shifts, the precision that can be achieved with respect to changes in the refractive index depends on the sensitivity and the peak line width. Larger nanoparticles tend to have high sensitivities, but their peaks are broadened by multipolar excitations and radiative damping. The structure and size of nanosensors have an influence on the resonance peak and the peak width, therefore the figure of merit (FOM) is very important for evaluating the sensing ability of nanosensors. LSPR peaks are expressed by the reflectivity, which is defined as the minor reflectivity with respect to the position of LSPR peaks. The reflectivity can be measured by the detection system described above. A FOM obtained by dividing the sensitivity by the resonance line width is widely used to characterize nanosensors' sensing capabilities The LSPR spectrum responses to three different sensors were detected in this study. The RIS of various structures was determined independently by measuring the extinction spectra in a series of solvents by varying RI values, which is the common method of determining the RIS. The RI of the different solvents was measured using an Abbe refractometer at room temperature. The solvents used were (RI in parentheses) water (1.333), ethanol (1.361) and *n*-heptanes (1.386).

**Figure 5.**
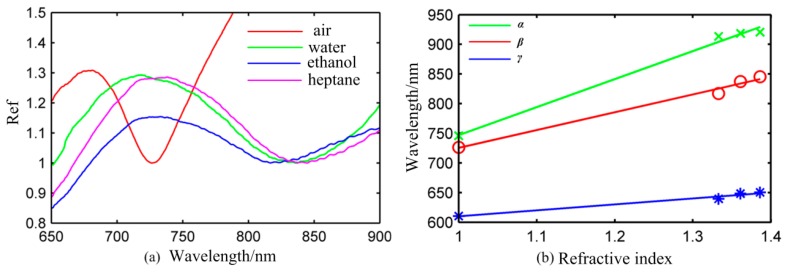
(**a**) The reflectivity values of nanosensor *β* in different surrounding media with respect to air, water, ethanol and *n*-heptane, respectively. All values of the reflection are applied normalization method; (**b**) LSPR peaks of *α* (crossed form), *β* (round form), *γ* (star form) in four mediums with respect to air, water, ethanol and *n*-heptane, respectively. Three lines are plotted according to the least square method (blue solid line, red solid line and green solid line).

According to the test results for the sensor chip *β* exposed to different solution presented in Figure 5a, the LSPR peak wavelength shifts remarkably from 726 nm to 845 nm when the refractive index varies from 1.000 to 1.386. When the refractive index of the medium varies slightly from 1.361 to 1.386, the LSPR peak also shifted by about 8 nm. This proved that the sensor chip *β* is exceedingly sensitive to refractive index changes and can be developed for highly sensitive biosensors. It is apparent that the peak of the sensor chip *β* was broadened in going from from air to solutions. This happens because peak width is related with the dielectric properties of the surrounding medium.

In order to investigate the overall relationship between resonant wavelength and the refractive index, LSPR peaks as a function of the refractive index for the sensor chip *α, β, γ* are depicted in Figure 5b. It can be easily found that the LSPR peak wavelength response is approximately linear to changes in refractive index of the surrounding medium.

Therefore, the refractive index sensitivity m of a particular nanoparticle type is usually reported in nanometers of peak shift per refractive index unit (nm/RIU), that is:

(2) m=dλ/dn

As shown in Figure 5b, although the plasmon resonance wavelength is not strictly linear to the index of refraction, it is linear to a good approximation over small ranges of n (R^2^ > 0.95). From the linear regressions of Figure 5b, we are able to calculate the bulk RIS (wavelength) *m*_α_ = 472.17 nm/RIU *FOM_α_* = 2.95, *m*_β_ = 300.16 nm/RIU *FOM_β_* = 2.5 and *m*_γ_ =100.21 nm/RIU *FOM_γ_* = 1.87. Obviously, the *α* type sensor is the most sensitive among the three types of sensors. As to the *α, β* type sensor, the resonance peak is blue-shifted in air when the thickness of Au shell decreases. The surface charges in the gold film provide the restoring force. The increase in the thickness of Au film leads to enhanced surface charge strength and increased restoring force. The higher plasmon energy caused by increased restoring force leads to the blue-shift of the dipole resonance. The thickness of the Au shell which was considered as an essential parameter to determine specific LSPR properties can be modulated to meet the needs of different sensing applications in practice. For the *γ* type sensor, the introduction of Au film leads to a blue-shift of the resonance peak. This can be interpreted the coupling of delocalized plasmon and leads to the higher plasmon energy. The PS spheres are covered with Au shells and Au film, which improve the stability of the nanosensors. Note that, although the *α* type sensor is very sensitive to the localized field from above data, it is not clear that how far the sensitive region occurs or where the highly sensitive region is. Therefore, it is necessary to study the decay length of the nanosensors for obtaining the highly sensitive region.

### 3.3. The Decay Length of the Nanosensors

If the nanosensors are to be successfully used as biosensors, we must determine the decay length to achieve high sensitivity when binding events occur in the decay length. Since the field decay length of the nanosensors is very short, the sensitivity will decrease when the size of the biomolecule is out of that field, so the decay length is quite important to estimate the sensitivity of the nanosensors. Because polyelectrolyte multilayers are simple, reproducible and have a highly controlled thickness polyelectrolyte multilayers were used to determine the decay length. Polyelectrolyte multilayers were deposited on the surface of Au film as described above and the corresponding extinction spectra were measured after the binding of each bilayer. The decay length of three nanosensors with different structures and parameters were measured according to Equation (1) in this study. The LbL assembly was carried out until the plasmon peak wavelength and intensity remained stable. The thickness of the layer was assumed to be 2.1 nm according to the measurement of the same multilayer system by Kedem [20].

**Figure 6.**
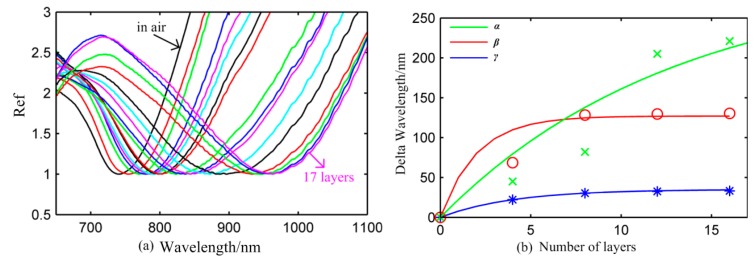
(**a**) The plasmon resonant peak and reflectivity of the *α* type nanosensor in air; (**b**) The delta peak wavelength of three nanosensors as a function of number of layers. The fitting curve of *α, β* ,*γ* sensors (green, red and blue solid line, respectively).

Figure 6a is the reflectivity value of the *α* type nanosensor in air when the PAH/PSS bilayer was continuously absorbed on the surface of the nanosensor. The left black solid line is the reflectivity of that without the bilayer in air. The right purple solid line is the reflectivity of the *α* type sensor which absorbed 17 layers in air. The LSPR peaks shifts remarkably from 740 nm to 965 nm with the increase in the number of bilayers. The LSPR peak shows a tendency to stabilize when the *α* type nanosensor absorbed 17 layers of the PAH/PSS bilayer. The result shows that the delta wavelength of three nanosensors decreases with the increase in number of layers. For each sensor, four sets of data corresponding to the fourth, eighth, twelfth, sixteenth layer were selected to make fit an exponential curve according to Equation (1) as shown in Figure 6b. The values of m and l are shown in Table 1.

The RIS data have been measured according to two methods as shown in Table 1. The bulk RIS values of the three nanosensors have more sensitivity than the m measured by LbL assembly. However, the bulk RIS and m have good agreement with the tendency that the α type nanosensor has the best and the γ type nanosensor has the worst sensitivity in air. The α type nanosensor has great advantages not only in RIS, but also in the decay length among three nanosensors, indicating its potential for small molecule detection and improving the limit of detection.

### 3.4. The Detection of Atrazine by the Core-Shell Nanosensor

It has been proved that the three nanosensors can be used for LSPR sensing. Atrazine antigen and antibody were chosen to test the sensing ability of the three nanosensors [21]. After the three core-shell nanosensors were treated for 12 h by PBS buffer solution, the surface of the *α, β* nanosensors were broken. That is to say, the stability of the *α, β* nanosensors is poor, which is caused by the weak binding force between the nanoparticles and glass. To achieve higher stability for the α type sensor, the glass slide can be treated by EDA and carboxylic polystyrene spheres can be used. Carboxylic polystyrene spheres react with EDA and the PS become linked with the glass slide by amide bonds. This method can improve the stability of the α type sensor. However, the nanoparticles of the γ nanosensor were closed in Au shell and film, which constituted a whole, so it was very stable, so the *γ* nanosensor was used for the detection of atrazine.

**Figure 7.**
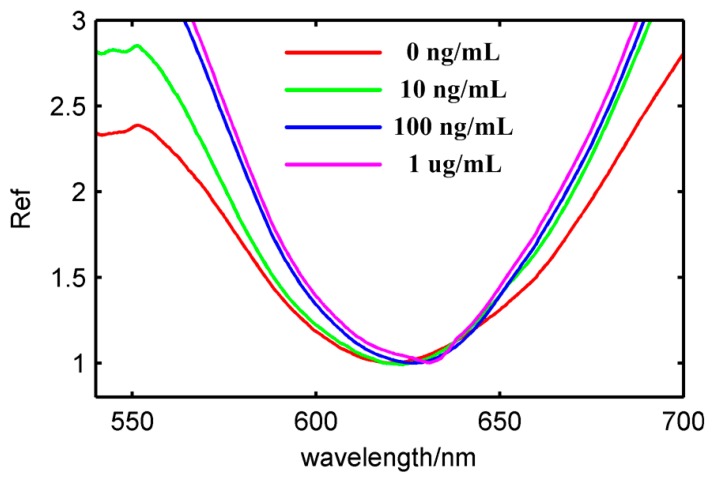
The reflectivity value of the atrazine antibody in solutions with different atrazine concentrations of 10 ng/mL (green solid line), 100 ng/mL (blue solid line), 1 μg/mL (purple solid line); the red solid line is the reflectivity value of atrazine antibody fixed in core-shell nanosensors.

As shown in Figure 7, atrazine antibody reacted with atrazine solutions with different concentration, which were 10 ng/mL, 100 ng/mL and 1 μg/mL, respectively. The resonance wavelength shifted 1.5 nm, 3 nm, 5 nm, respectively. The antibody-antigen reaction formed a new molecule and led to an increase of the refractive index on the surface of Au shell. The atrazine concentration can be tested by the LSPR peak response shown in Figure 7. Results show that the *γ* nanosensor can trace the content of atrazine at 10 ng/mL. Even though there are many chemical procedures for biofunctionalization, these core-shell nanosensors have good potential with higher sensitivity and applicability through the use of plasmonic biosensors.

## 4. Conclusions

We have presented a systematic study of the fabrication and the LSPR sensing performance of some new nanosensors. The core-shell nanosensors used were fabricated by assembly and ion sputtering technology and exhibited good repeatability and stability. The RIS was measured by two methods: one is the effect of the change of the surrounding medium on the refractive index; the other is using the polyelectrolyte LbL assembly. The decay length was obtained using polyelectrolyte LbL assembly and fitting to a model of an exponentially decaying surface plasmon field. Results show that the RIS and the decay length, which are important parameters relevant to sensing applications, could be tuned by varying the thickness of the Au shell for core-shell nanosensors. The core-shell nanosensors with Au film can monitor the content of atrazine at concentrations as low as 10 ng/mL. To meet different sensing demands such as biorecognition and concentration dectection, core-shell nanosensors should be further optimized to obtain good sensing performance.

## Figures and Tables

**Table 1.** The parameters describing sensing performance for the three nanosensors.

	**Bulk RIS nm/RIU**	**FOM**	**m nm/RIU**	**l/nm**
α	472.17	2.95	300	26
β	300.13	2.50	127	10
γ	100.21	1.87	35	8
